# Expression of Kisspeptin 1 in the Brain of the Adult Sea Lamprey *Petromyzon marinus*

**DOI:** 10.3390/life11111174

**Published:** 2021-11-03

**Authors:** Daniel Sobrido-Cameán, Luis Alfonso Yáñez-Guerra, Alexandre Deber, María Celina Rodicio, Antón Barreiro-Iglesias

**Affiliations:** 1 Department of Functional Biology, CIBUS, Faculty of Biology, Universidade de Santiago de Compostela, 15782 Santiago de Compostela, Spain; ds918@cam.ac.uk (D.S.-C.); xandredl@hotmail.com (A.D.); mcelina.rodicio@gmail.com (M.C.R.); 2 Living Systems Institute, University of Exeter, Exeter EX4 4QD, UK; L.Yanez-Guerra@exeter.ac.uk

**Keywords:** kisspeptin, hypothalamus, in situ hybridization, lamprey, reproduction

## Abstract

Kisspeptin peptides play major roles in the regulation of reproduction and puberty onset in mammals. While most mammals only have one kisspeptin gene, other jawed vertebrates present two or three genes. Recent data also revealed the presence of two genes in lampreys (jawless vertebrates). However, apart from gene sequence data, there is almost no information on the kisspeptinergic system of lampreys. Here, we report phylogenetic and cluster-based analyses showing that the duplication of the ancestral kisspeptin gene occurred before the separation of jawless and jawed vertebrates. We also studied the expression of the kisspeptin transcripts in the brain of post-metamorphic juveniles and upstream migrating adult sea lampreys. Our in situ hybridization results revealed expression of kisspeptin 1 in hypothalamic neurons, which indicates that the hypothalamic expression of kisspeptins is an ancestral character in vertebrates. We also observed the presence of kisspeptin 1 expressing neurons in the paratubercular (posterior tubercle) nucleus of the diencephalon. This is the first description of the presence of kisspeptin 1 expressing neurons in this brain region in any vertebrate. We did not detect expression of kisspeptin 2 in the juvenile or adult sea lamprey brain with in situ hybridization. Our data provides an anatomical basis to study the role of kisspeptin 1 in the hypothalamic-pituitary system of lampreys and the contribution of diencephalic kisspeptinergic neurons to different circuits of the lamprey brain.

## 1. Introduction

Kisspeptin (Kiss) neuropeptides are part of the RFamide family [1], and they act by binding G-protein coupled receptors that are named GPR54 or *Kiss*R [2]. Functional *Kiss* genes are present in most vertebrates (apart from birds, which only present a *Kiss* pseudogene [3]) and vertebrate *Kiss* genes are derived from an ancestral *Kiss* gene that during evolution underwent several duplications followed by gene losses [4]. Actinopterygians have 2 *Kiss* genes (*Kiss*1 and *Kiss*2), amphibians, like *Xenopus*, have 3 (*Kiss*1*a*, *Kiss*1*b* and *Kiss*2) and most mammals only have the *Kiss*1 gene [4]. The recent identification of *Kiss1* and *Kiss*2 genes in the sea lamprey suggested that the duplication of the ancestral *Kiss* gene occurred before the separation of jawless and jawed vertebrates [4,5]. However, the possibility that this is a linage-specific duplication in lampreys should not be excluded and new phylogenetic analyses that include the sea lamprey *Kiss* precursors and the third *Kiss* genes from other vertebrates (e.g., *Xenopus*) are needed to differentiate between these two scenarios. Mature *Kiss* peptides play a major role in the regulation of reproduction in mammals, with *Kiss*1 expressing hypothalamic neurons playing an essential role in the regulation of puberty onset and the control of gonadotropin releasing hormone (GnRH) and gonadotropin secretion [6].

Lampreys occupy an interesting phylogenetic position to infer ancestral characters fixed prior to the divergence of jawless and jawed vertebrates or to identify lineage specific diversifications. Sea lampreys, *Petromyzon marinus* L, have a complex life cycle with a filter-feeding larval period lasting several years in the river followed by a transformation into young adults (juveniles) that migrate to the sea before returning to the river to breed and die. Work in the last decades has provided interesting genetic, neuroanatomical, and functional data on the hypothalamic-pituitary system of lampreys and on many of the hormonal/neuropeptidergic (e.g., GnRHs) systems controlling the life cycle and reproduction of lampreys (for a review see [7]). However, the kisspeptinergic system has not yet been thoroughly studied in lampreys and there is no information on the expression or role of kisspeptins in these animals. Only one study has reported the inhibitory effect of lamprey *Kiss*1 mature peptides on the expression of the luteinizing hormone ß subunit in primary cultures of eel pituitary cells [8]. The recent identification of *Kiss* genes in the sea lamprey genome ([3]; see above) provides a basis to study their expression and the organization of the kisspeptinergic system in lampreys, which will increase our knowledge on the evolution of this system and the physiological control of reproduction in vertebrates.

Here, we performed phylogenetic and cluster-based (CLANS) analyses of the vertebrate *Kiss* precursors and aimed to study the expression of the 2 *Kiss* transcripts in the brain of sea lampreys, both in young post-metamorphic juveniles and in upstream migrating adults. Our in situ hybridization data revealed the presence of hypothalamic neurons expressing *Kiss1*. This indicates that hypothalamic *Kiss* expression is an ancestral character for all vertebrates and that *Kiss1* might also play a role in the control of GnRH release in lampreys. Our anatomical data provides a basis for future functional studies on the kisspeptinergic system in lampreys.

## 2. Materials and Methods

### 2.1. Alignment of the Kiss1 and Kiss2 Sea Lamprey Precursor Sequences with Kiss Precursor Sequences from other Chordates and Phylogenetic Analyses

**Figure 1:**
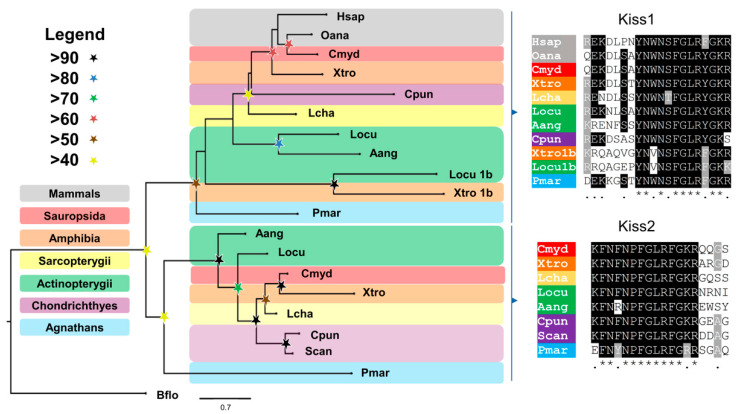
Maximum-likelihood tree and alignment of the C-terminal region of Kiss precursors showing relationships of these precursor proteins from selected chordate species. The percentages of replicate trees in which the associated taxa clustered together in the approximate Bayesian branch support test (aBayes 1000 replicates) are shown with stars as explained in the key. In the right side, alignments of the C-terminal region, including the mature peptides of *Kiss*1 and *Kiss*2 precursors are shown. The conserved residues are highlighted, with conservation in more than 70% of sequences shown in black (asterisks indicate a 100% of conservation in a specific residue) and with conservative substitutions shown in grey. Species names in the tree and alignment are as follows: Hsap (*Homo sapiens*), Oana (*Ornithorhynchus anatinus*), Cmyd (*Chelonia mydas*), Xtro (*X. tropicalis*), Lcha (*Latimeria chalumnae*), Locu (*Lepisosteus oculatum*), Aang (*A. anguila*), Cpun (*Chiloscyllium punctatum*), Scan (*Scyliorhinus canicula*), Pmar (*P. marinus*).

The *Petromyzon marinus Kiss* cDNA sequences were identified using the human *Kiss* precursor as a query (GenBank accession number: NP_002247.3) and performing TBLASTn against the *P. marinus* TSA database. The sea lamprey *Kiss1* and *Kiss2* cDNA sequences were translated to the corresponding amino acid sequence using the ExPASy website translate tool (https://web.expasy.org/translate/, accessed on 4 October 2021). To investigate the relationship of the sea lamprey *Kiss*1 and *Kiss*2 precursors to other Kiss precursors, sequences from other species were obtained from GenBank and used to perform a phylogenetic analysis using the maximum-likelihood method (see Supplementary Files S1 and S2 for a list of the precursors). The amino acid sequences of the sea lamprey Kiss precursors were aligned with Kiss precursors from other species using MAFFT (G-INS-I iterative; see Supplementary File S3 for the sequences used for the tree and the accession numbers of the sequences). The alignment was trimmed using TrimAl in the automated option [9]. The tree was constructed using PhyML [10]. The percentage of replicate trees in which the associated taxa clustered together in the approximate Bayesian branch support test (aBayes, 1000 replicates) are shown with stars in Figure 1. The substitution model used was the LG + G4 model. The tree was rooted using figtree v1.4.4 and the tree was exported using this tool. The raw tree data is available as the Supplementary File S4.

Finally, the alignment with no trimming was used to highlight the conserved residues using the software BOXSHADE (www.ch.embnet.org/software/BOX_form.html, accessed on 4 October 2021) with 70% conservation as the minimum for highlighting. The whole precursor highlighting is available in the Supplementary Files S1 and S2. The regions containing the mature peptide were trimmed and used to show the conservation of the Kiss mature peptides belonging to the *Kiss*1 and *Kiss*2 clusters of sequences. Finally, the sequences were highlighted in phylum-specific colours: mammals (gray), sauropsids (red), amphibians (orange) lobe-finned fishes (yellow), ray-finned fishes (green), cartilaginous fishes (purple), and agnathans (blue).

To further investigate the relationship of the sea lamprey Kiss precursors with other vertebrate precursors we performed a cluster-based analysis (CLANS). Using the same sequences for the phylogeny an all-versus-all analysis was performed using CLANS [11] with the matrix BLOSUM62 as the scoring matrix. Sequences were clustered with an e-value cut-off of 1 × 10^−8^ to identify clusters. The clustering was collapsed to 2D to enable the generation of the diagram. The clustering analysis is available as Supplementary Figure S1.

### 2.2. Animals

Larvae (*n* = 8), downstream migrating young (post-metamorphic juveniles; *n* = 3) and upstream migrating (*n* = 3) adult sea lampreys, *Petromyzon marinus* L., were used in this study. Larvae and downstream migrating young adults were collected from the River Ulla (Galicia, Spain) with permission from the *Xunta de Galicia*. Upstream migrating adults were acquired from local suppliers. Before all experiments, animals were anesthetized with 0.1% tricaine methanesulfonate (Sigma, St. Louis, MO, USA) in fresh water and killed by decapitation. Information on the sex of juvenile/adult animals was not available since we used a collection of brains already stored in the laboratory. All experiments were approved by the Bioethics Committee at the University of Santiago de Compostela and the *Consellería do Medio Rural* of the *Xunta de Galicia* (Ref. 15012/2020/011) and were performed in accordance with European Union and Spanish guidelines on animal care and experimentation.

### 2.3. Cloning and Sequencing of the Sea Lamprey Kiss1 and Kiss2 cDNAs

Total RNA was obtained from the central nervous system (brain and spinal cord) of the larval samples by using the TriPure reagent (Roche, Basel, Switzerland). cDNA synthesis reaction from total RNA was catalysed with Superscript III reverse transcriptase (Invitrogen, Waltham, MA, USA) using random hexamer primers (Invitrogen). For polymerase chain reaction (PCR) amplification, specific primers (*Kiss*1: forward: 5′-GGGACTGACGGTCGTGACAT-3′, reverse: 5′-CCCAAAACGCAACCCAAACG-3′; *Kiss*2: forward: 5′-GGAGGAGATCGTCACCGGG-3′, reverse: 5′-GCAGGCCGAAGGGGTTGTA-3′) were designed based on the *P. marinus* L *Kiss1* and *Kiss2* transcript precursor sequences (GenBank accession numbers KT202350.1 and KT202356.1 [12]). The amplified fragments were cloned into pGEM-T easy vectors (Promega, Madison, WI, USA) and sequenced by GATC Biotech (Cologne, Germany). This confirmed that we cloned a 364 bp cDNA sequence of the sea lamprey *Kiss*1 and a 209 bp cDNA sequence of the sea lamprey *Kiss*2.

### 2.4. In Situ Hybridization on Tissue Sections

Templates for riboprobe generation were prepared by PCR amplification of the cloned *Kiss1* and *Kiss2* cDNA fragments using the primers mentioned above, although in this case the reverse primers included the sequence of the T7 promoter (TAAGCTTTAATACGACTCACTATAGGGAGA). For the generation of sense probes, the sequence of the T7 promoter was included in the forward primers. Digoxigenin (DIG)-labelled riboprobes were synthesized using the amplified fragments as templates and a T7 RNA polymerase (Nzytech, Lisbon, Portugal).

In situ hybridization was performed as previously described for other neuropeptide riboprobes in the sea lamprey [13,14,15]. Briefly, the brains/rostral spinal cords of young and mature adults were fixed by immersion overnight in 4% paraformaldehyde in phosphate-buffered saline (PBS) at 4 °C. They were cryoprotected with 30% sucrose in PBS, embedded in Tissue Tek (Sakura, Torrance, CA, USA), frozen in liquid nitrogen-cooled isopentane, and cut serially on a cryostat (14 µm thickness) in transverse planes. Sections were mounted on Superfrost^®^ Plus glass slides (Menzel, Braunschweig, Germany). The sections were incubated with the DIG-labelled antisense riboprobe (1 µg/mL) at 70 °C overnight in hybridization mix and treated with RNAse A (Sigma) in the post-hybridization washes. Then the sections were incubated with a sheep anti-DIG antibody conjugated to alkaline phosphatase (1:2000; Roche, Basel, Switzerland) overnight at 4 °C. The colorimetric reaction was conducted using BM Purple (Roche) at 37 °C until the signal was clearly visible. The sections were mounted using Mowiol^®^ (Sigma). No staining was observed in the sections incubated with the sense probes (Supplementary Figure S2A).

### 2.5. Imaging and Figure Preparation

Photographs of brain sections were taken with an Olympus microscope (AX-70; Provis, Olympus, Tokyo, Japan) equipped with a digital camera (Olympus DP70). Schematics and plates of photomicrographs shown in Figure 1 were generated with Adobe Photoshop CS6 (Adobe, San Jose, CA, USA).

## 3. Results

### 3.1. Sequence Analyses

Based on an alignment of 20 selected Kiss precursor protein sequences from different gnathostome species and the agnathan *P. marinus*, a phylogenetic reconstruction was performed using the maximum-likelihood method. The tree was rooted using the sequence of a Kiss precursor from *Branchiostoma floridae*. Most of the species included in the analyses have two Kiss precursors, *Kiss*1 and *Kiss*2. The exceptions were *Xenopus tropicalis* and *Lepisosteus oculatus*, in which there are three different Kiss precursors (the third one named as *Kiss*1b). As shown in Figure 1, the phylogenetic reconstruction revealed the presence of only two branches constituting two clusters of Kiss precursors, the *Kiss*1 and *Kiss*2 clusters. The third precursor identified in *X. tropicalis* and *L. oculatus* form part of the *Kiss*1 branch of the tree, suggesting that they represent a recent duplication of *Kiss*1 in these species. It is important to notice that the node supporting the two main branches of *Kiss*1 and *Kiss*2 displays a low branch support value (>40%). Thus, to further test this hypothesis, a cluster-based analysis was performed with CLANS. The cluster analysis revealed a similar pattern to the one obtained in the phylogenetic analysis, with two main clusters produced by the sequences. One cluster corresponding to the *Kiss*1 precursors and the other one containing the *Kiss*2 sequences. The *Kiss*1b precursor sequences from *L. oculatus* and *X. tropicalis* show connections only to the *Kiss*1 cluster.

An alignment of the predicted mature peptides shows that there is a high degree of conservation of the mature peptides in vertebrates. The mature *Kiss*1-10 peptides (including the duplicated ones from *X. tropicalis* and *L. oculatus*) have the consensus sequence YNWNSFGLRYG, while the *Kiss*2–10 peptides have the consensus sequence FNFNPFGLRFG. A comparison of the *P. marinus Kiss*1 peptide against the consensus sequence shows that there is only 1 mutation in the C-terminal region, with the exchange of the (Y-amide) tyrosine-amide for an (F-amide) phenylalanine-amide (Figure 1). In the case of the *Kiss*2 peptide, there is one mutation in the N-terminal region, with the exchange of the second (F) phenylalanine for a (Y) tyrosine (Figure 1).

### 3.2. Expression of the Kiss1 and Kiss2 Transcripts in the Adult Sea Lamprey Brain

**Figure 2:**
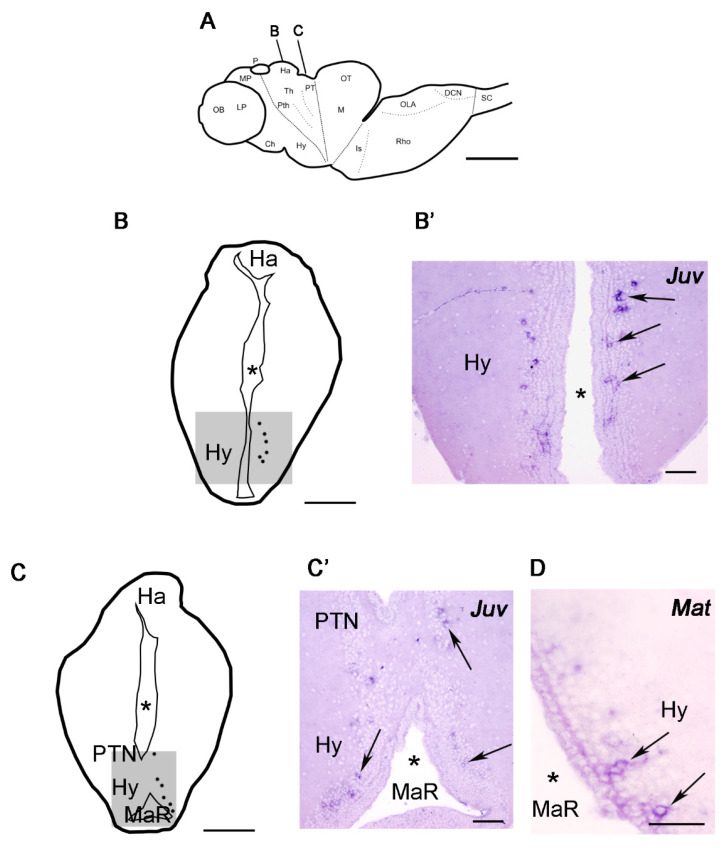
Schematic drawings of sections of the sea lamprey brain showing the distribution of cells expressing *Kiss*1 (**A**,**B**,**C**) and photomicrographs of in situ hybridized transverse sections showing the expression of the *Kiss*1 mRNA in the brain of juvenile (**B**’,**C**’) and mature (**D**) sea lampreys. (**A**): Figurine representing a lateral view of the brain. The planes of the transverse sections in B and C are indicated. (**B**,**C**): Cells are represented as dots on the right side of the sections. Anatomical regions are presented on the left side. (**B’**,**C’**): Photomicrographs of transverse sections of the brain of post-metamorphic juvenile (Juv) sea lampreys. (**D**): Photomicrograph of a transverse section of the brain of a mature (Mat) upstream migrating adult sea lamprey. Asterisks indicate the ventricle. The arrows indicate *Kiss*1 expressing cells. The approximate regions of photomicrographs are represented with shadow squares in the transverse schematic drawings. Scale bars: A, B and C 1 mm; B’, C’ and D 100 µm. Abbreviations: Ch, optic chiasm; DCN, dorsal column nucleus; Ha, habenula; Hy, hypothalamus; Is, Isthmus; LP, lateral pallium; M, mesencephalon; MaR, mamillary recess; MP, medial pallium; OB, olfactory bulbs; OLA, octavolateralis area; OT, optic tectum; P, pineal organ; PT, pretectum; Pth, prethalamus (ventral thalamus); PTN, posterior tubercle nucleus (paratubercular nucleus); Rho, rhombencephalon; SC, spinal cord; Th thalamus (dorsal thalamus).

As indicated in the materials and methods section, we cloned partial sequences of the sea lamprey *Kiss*1 and *Kiss*2 cDNAs and used them as templates to generate specific riboprobes that were used for in situ hybridization in transverse sections of the sea lamprey brain. We did not detect expression of *Kiss*2 transcripts in the brains of post-metamorphic juveniles or upstream migrating adults (Supplementary Figure S2B). In the case of *Kiss*1, we did not detect any differences in the distribution and location of *Kiss*1-expressing cells in post-metamorphic juveniles and upstream migrating adults; therefore, *Kiss*1 in situ hybridization results are described together for both stages (Figure 2).

*Kiss*1 expression was mainly found in the hypothalamus, with numerous *Kiss*1-expressing neurons being present in the tuberal (Figure 2A–B’) and mamillary (Figure 2A,C,C’) regions of the hypothalamus. Interestingly, we also observed the presence of a few *Kiss*1 expressing cells in the paratubercular (posterior tubercle) nucleus (Figure 2A,C,C’) of the diencephalon, which is located dorsally and caudally to the hypothalamus (in the basal part of prosomeres 2 and 3).

## 4. Discussion

In 2009, Felip et al. identified two different *Kiss* precursors in *P. marinus* [5]. Based on an unrooted phylogeny, the same authors proposed the hypothesis that only 2 Kiss precursors clusters exist in vertebrates, the *Kiss*1 and *Kiss*2 clusters [5]. However, sequences of species with more than two Kiss precursors were not included in their analysis. For example, from *X. tropicalis*, only two of the three Kiss precursors were included in the phylogeny. Later that year, Lee et al. described the presence of three different Kiss precursors in *X. tropicalis* [4]. Based on alignments of the mature peptides derived from the three *X. tropicalis* precursors, Lee et al. proposed that the third Kiss precursor from *X. tropicalis* was a duplication of the *Kiss*1 precursor and named it *Kiss*1b. However, they did not perform a phylogeny to confirm this hypothesis [4]. Here, in our phylogeny we included the sequences of a third Kiss precursor from *X. tropicalis* and *L. oculatus* and generated a rooted tree. Importantly, the node supporting the two branches of *Kiss*1 and *Kiss*2 precursors is supported by a low branch support. It is not uncommon to obtain low support values in neuropeptide phylogenies. This is due to the short sequences of neuropeptide precursors and because neuropeptides evolve at non-homogeneous rates at different regions of the peptides [16]. For example, the regions encoding the mature peptides, the signal peptide and cleavage sites evolve under strong pressures of selection, while the rest of the precursor is highly evolvable [16]. Thus, to further test the relationship of Kiss precursors, a CLANS analysis was also performed. This analysis produced a very similar result to the one observed in the phylogeny with two main clusters, one corresponding to the *Kiss*1 and the second one corresponding to the *Kiss*2 precursors. Together, our results confirm the hypothesis from previous studies. First, there are only two clades of Kiss precursors, the *Kiss*1 and *Kiss*2 clades, and *P. marinus* has a precursor in each branch of the phylogeny or in each cluster of the CLANS analysis. Second, the precursors from *X. tropicalis* and *L. oculatus* are the product of a recent duplication. Our phylogenetic and cluster-based analyses indicate that the duplication of the ancestral Kiss precursor occurred before the separation of jawless and jawed vertebrates.

This is the first study to report the expression of the *Kiss*1 gene in the brain of any jawless vertebrate. Our results indicate that *Kiss* hypothalamic expression is an ancestral character of all vertebrates since *Kiss*1 or *Kiss*2 genes are also expressed in the hypothalamus of different jawed fish (medaka: [17,18,19]; zebrafish: [18,19,20]; striped bass: [21]; European sea bass: [22,23]), reptiles (Chinese alligator: [24]), amphibia (Western clawed frog: [4]) or mammals (mouse: [25]). Our results revealing hypothalamic expression also suggest that Kiss1 could play a role in the regulation of the release of GnRH (the three lamprey GnRHs are expressed in the hypothalamus of both post-metamorphic juveniles and upstream migrating adults [26]) and pituitary glycoprotein hormones [7] in lampreys as in other vertebrates. Expression of *Kiss1* in post-metamorphic juveniles and in upstream migrating adults suggests a relationship with key events in sexual maturation/breeding. Functional work should attempt to decipher the role of *Kiss*1 peptides in the hypothalamic-pituitary system of lampreys and confirm its possible role in the regulation of GnRH release.

Our in situ hybridization analyses also revealed the expression of *Kiss*1 transcripts in a few cells of the paratubercular (posterior tubercle) nucleus of the diencephalon. As far as we are aware, this is the first description of the presence of *Kiss*1 neurons in this region in any vertebrate, although expression of *Kiss*2 has been also reported in the zebrafish posterior tuberculum [9,20,27]. Despite its diencephalic location, it has been suggested that this nucleus is homologous of the substantia nigra/ventral tegmental area of mammals [28] since it contains a cluster of dopaminergic cells that projects to the striatum [26,29]. Tract-tracing studies in lampreys have also revealed projections from posterior tubercle dopaminergic cells to the optic tectum [29], rhombencephalon [30] and rostral spinal cord [31,32]. Future studies should try to determine whether dopaminergic cells of this region also express *Kiss*1 (as it happens with dopaminergic cells in the rostral periventricular area of the third ventricle in mice [25]) and the possible role of *Kiss*1 mature peptides in striatal, tectal, rhombencephalic and/or spinal cord circuits in lampreys.

We could not detect expression of *Kiss*2 transcripts in the juvenile or adult lamprey brain. This negative result could reflect a true lack of *Kiss*2 expression or could be related to insufficient sensitivity of the in situ hybridization protocol. Unfortunately, with our current data we cannot differentiate between these two scenarios. The detection of the *Kiss*2 cDNA by RT-PCR from larval central nervous system samples suggests the possibility that *Kiss*2 could also be expressed in the adult central nervous system but at very low levels, impeding its detection by conventional in situ hybridization (which is less sensitive than RT-PCR methods). The use of other histological or biochemical methods in the future might help in determining whether *Kiss*2 is expressed in the sea lamprey brain.

## 5. Conclusions

In conclusion, the expression of *Kiss* genes in the hypothalamus appears to be an ancestral character for all vertebrates. Interestingly, in lampreys, *Kiss*1 is also expressed in the paratubercular nucleus, which reveals an important difference in the organization of the kisspeptinergic system in comparison to mammalian species.

## Data Availability

Data is contained within the article, and materials can be requested from the authors upon reasonable request.

## Supplementary Materials

The following are available online at https://www.mdpi.com/article/10.3390/life11111174/s1. Supplementary Figure S1: Cluster-based analysis of the relationships of Kiss precursors in selected species. Nodes correspond to the precursors and are color-coded according to the key. Supplementary Figure S2: Figurine representing a lateral view of the sea lamprey brain (A) and photomicrographs of in situ hybridized transverse sections showing the lack of positive signal of the *Kiss*1 sense probe (negative control; B) and the lack of detectable expression of the *Kiss*2 mRNA using the *Kiss*2 anti-sense riboprobe in the brain of juvenile (Juv) sea lampreys (C). The planes of the transverse sections in B and C are indicated in A. Scale bars: A 1 mm, B and C 200 µm. Abbreviations: Ch, optic chiasm; DCN, dorsal column nucleus; Ha, habenula; Hy, hypothalamus; Is, Isthmus; LP, lateral pallium; M, mesencephalon; MaR, mamillary recess; MP, medial pallium; OB, olfactory bulbs; OLA, octavolateralis area; OT, optic tectum; P, pineal organ; PT, pretectum; Pth, prethalamus (ventral thalamus); PTN, posterior tubercle nucleus (paratubercular nucleus); Rho, rhombencephalon; SC, spinal cord; Th thalamus (dorsal thalamus). Supplementary File S1: *Kiss*1 alignments. Supplementary File S2: *Kiss*2 alignments. Supplementary File S3: Fasta file with the sequences used for the tree construction. Supplementary File S4: Nhx file with raw phylogenetic tree data.
