# Supplementary material for: Expression of Kisspeptin 1 in the Brain of the Adult Sea Lamprey Petromyzon marinus
Source: Life (Basel). 2021 Nov 3;11(11):1174. doi: 10.3390/life11111174 (PMC8624340; doi:10.3390/life11111174)
Supplement: Supplementary file 1 [file life-11-01174-s001.zip › life-1430285-supplementary for conversion/life-1430285-supplementaryFigures.docx]

Supplementary materials Figures


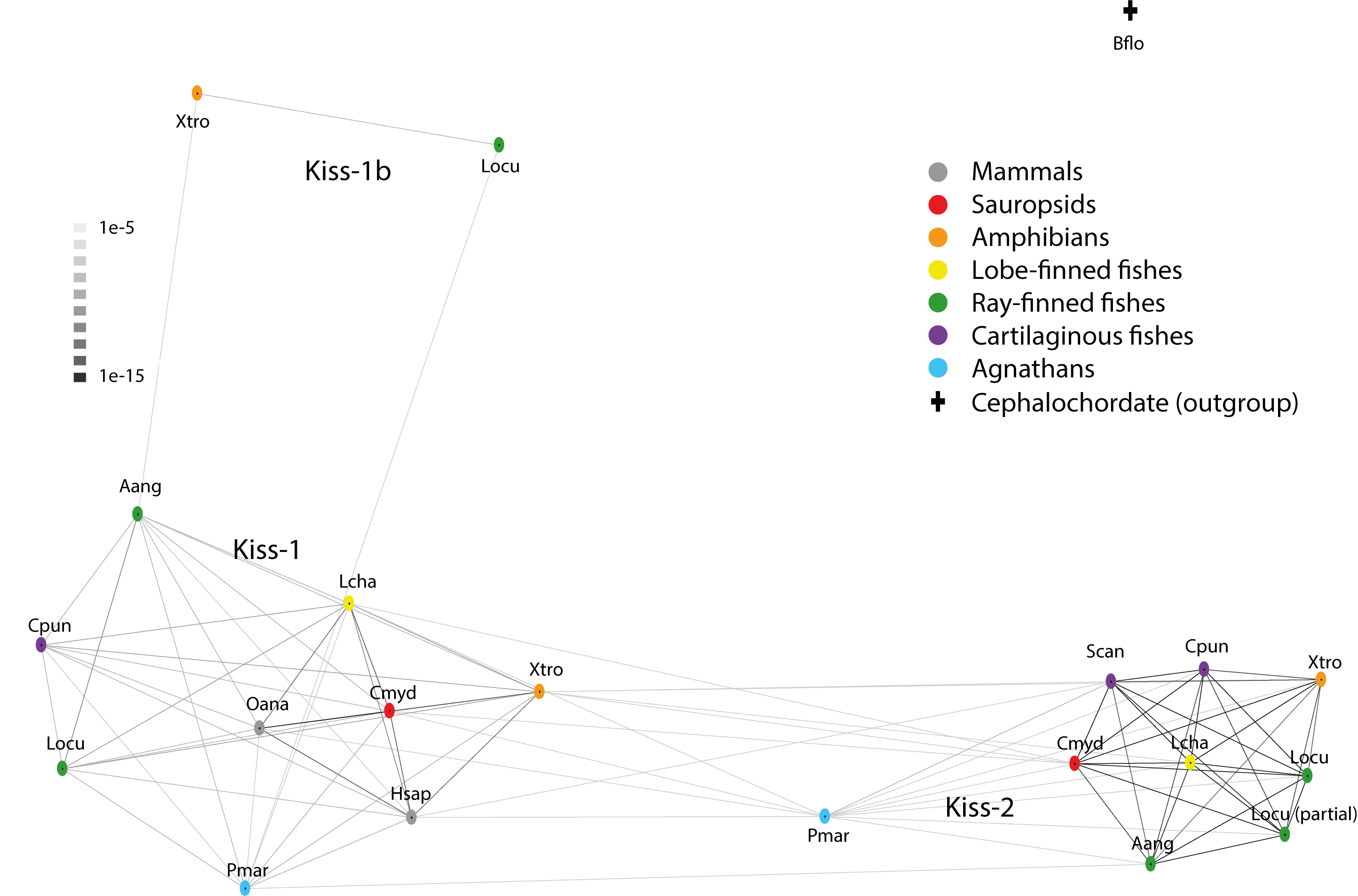


**Figure S1.** Cluster-based analysis of the relationships of Kiss precursors in selected species. Nodes correspond to the precursors and are color-coded according to the key.


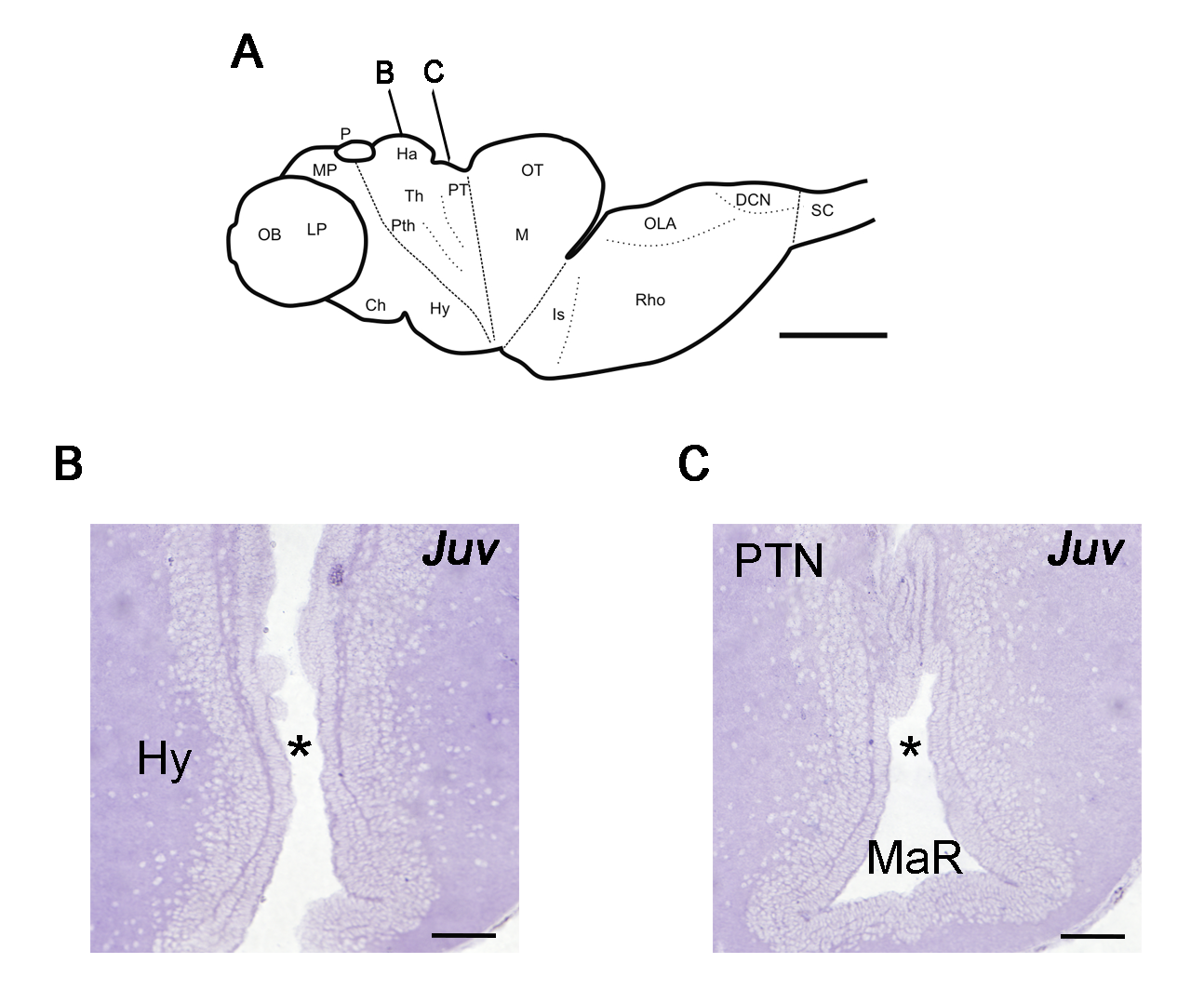


**Figure S2.** Figurine representing a lateral view of the sea lamprey brain (A) and photomicro-graphs of in situ hybridized transverse sections showing the lack of positive signal of the Kiss1 sense probe (negative control; **B**) and the lack of detectable expression of the Kiss2 mRNA using the Kiss2 anti-sense riboprobe in the brain of juvenile (Juv) sea lampreys (**C**). The planes of the transverse sections in B and C are indicated in A. Scale bars: A 1 mm, B and C 200 µm. Abbrevia-tions: Ch, optic chiasm; DCN, dorsal column nucleus; Ha, habenula; Hy, hypothalamus; Is, Isth-mus; LP, lateral pallium; M, mesencephalon; MaR, mamillary recess; MP, medial pallium (pre-thalamic eminence of Pombal et al., 2009); OB, olfactory bulbs; OLA, octavolateralis area; OT, optic tectum; P, pineal organ; PT, pretectum; Pth, prethalamus (ventral thalamus); PTN, pos-terior tubercle nucleus (paratubercular nucleus); Rho, rhombencephalon; SC, spinal cord; Th thalamus (dorsal thalamus).
