# Supplementary material for: Expression of Kisspeptin 1 in the Brain of the Adult Sea Lamprey Petromyzon marinus
Source: Life (Basel). 2021 Nov 3;11(11):1174. doi: 10.3390/life11111174 (PMC8624340; doi:10.3390/life11111174)
Supplement: Supplementary file 1 [file life-11-01174-s001.zip › life-1430285-supplementary for conversion/SupplementaryFile1.docx]

NP_002247_3 Hsap 1 ---------------------------------------------MNSLVSWQLL--LFLCATHFGEPLE
XP_028924153_1 Oana 1 ---------------------------------------------MSSLTSLLLL--LFLCAFPFGETVE
XP_027683240_2 Cmid 1 MASSGHVAGDLWLHHPLQEFFPSHLWISGSFGKYSTAEQGLGQPGMNPLSSMLLL--LFFSNAPFGEPTE
NP_001156331_1 Xtro 1 ---------------------------------------------MSSLC---LF--LFLLGIHLGRSDH
XP_005991747_1 Lcha 1 ---------------------------------------------MSPYIFLFLM--LFLKG-HLGEPLH
GCC34347_1 Cpun 1 ---------------------------------------------MNSEELHQAV--GFPCSSHL-----
SOR24334_1 Aang 1 ----------------------------------------------------------SMVTIRFGE---
XP_015197899_1 Locu 1 M---GHTGKEM----PCSP--------------------------LRTESTMLLLTMMLMMSVQLVEP--
XR_004403334_1 Pmar 1 ---------------------------------------------MRGLTVVTFLF-LVLCCDSFGKVVS
XP_015195061_1 Locu 1 ---------------------------------------------MRLPAISLLV--AAVCL--------
NP_001163986_1 Xtro 1 ---------------------------------------------MNLGGLSCFV--ICRLATDLQDFLT
consensus 1 . .. . . ..

NP_002247_3 Hsap 24 K-VASVGNSRPTG---------------QQLESLGLLAPG--------------------EQSLPCTERK
XP_028924153_1 Oana 24 K-FTPIQNPRFSGRR------------SRQLASLLEQTPGLWEPVRERTRGQWERTPGQWERTPPCLDKK
XP_027683240_2 Cmid 69 R-YLPLWSPRHTGDH------------FKHLAS-----------------------PAHWDQAIPCSESK
NP_001156331_1 Xtro 21 T--------------------------AKNTDELYSQVPG----------------KSQWLGSLLCPEKV
XP_005991747_1 Lcha 23 T-IVPIQTPVLTGEV------------LKAIAS------G----------------LLQKEESAPCLVQT
GCC34347_1 Cpun 19 --------------------------------------------------------------------RD
SOR24334_1 Aang 10 ---------------------------------------------------------------TRSLRYT
XP_015197899_1_Locu 36 -----------------------------------------------------------WAGHPQISSAS
XR_004403334_1 Pmar 25 F-YGFKESTKSGGGQ----LPGDVTDILREITSLL---EG--------------------TDGIVAFYDF
XP_015195061_1 Locu 16 ---------------------------------------G--------------------VQSADPSQFL
NP_001163986_1 Xtro 24 LFFIFFCNPEPTGGDGNLALTLDICDLIEVLYS---------------------------SSTPQNFPTM
consensus 71

NP_002247_3 Hsap 58 PAATARLSR------RGTSLSPPP-ES-SGS------PQQPGLSAPHS------------RQIPAPQGAV
XP_028924153_1 Oana 81 PGPEAAEQT------ARLALLCPP-EE-SPG------QVWRGLCPTQS------------QLVTGPQGGM
XP_027683240_2 Cmid 103 PSPGKAEPK------PVPPLLCKPQEE-SQA------QLGQGIHPARS------------RAVSVPQGSP
NP_001156331_1 Xtro 49 PTTRRAEQM------PVLSLLCRRKKSLSTG------HPWSTDSLLPS------------RSISAPEGEF
XP_005991747_1 Lcha 58 PQSRTQHPKML---RPLVKLFDLK-HG-SRP------RISRKIGLSLC------------KFNSSSLGVQ
GCC34347_1 Cpun 21 PSVITNSKD------PLYNLFRRQ-QALSWE------LC--IIKLILN------------KHNSRGAELD
SOR24334_1 Aang 17 PYTADEDPE-----IAAMRVLRQMSEELSTEAPPYPHRPAHWPTALANPLPGGAGLPRRSWWWYPEVPPQ
XP_015197899_1_Locu 47 PTVSGKKPE-----PGVQDILRRMSTTPPPGARLILPAAGKIPPALAS-LLFGSRFPRRGGWAQARPQPP
XR_004403334_1 Pmar 67 PGSGGSVDRAFMSPLHFYPMLRARMRSLPAS---------------------------------------
XP_015195061_1 Locu 27 P-------------LPLRTLRYRNVDSAPPP------LPVDAPGLLAS-------------------RLE
NP_001163986_1 Xtro 67 PQVTTNTSQ----------ILKNYKED-----------------LLAS------------------FFFQ
consensus 141 * .. . .

NP_002247_3 Hsap 102 LVQREKDLPNYNWNSFGLRFGKREAAPG-----------NHGRSAGR----------G
XP_028924153_1 Oana 125 LVEQEKDLSAYNWNSFGLRYGKRHAGVL------------KARLKIW-----------
XP_027683240_2 Cmid 148 PMEQEKDLSAYNWNSFGLRYGKRQAAIG------------EAKSSHR----------A
NP_001156331_1 Xtro 95 LVQREKDLSTYNWNSFGLRYGKRGSGSE------------NSKTKVW-----------
XP_005991747_1 Lcha 105 TVKRENDLSSYNWNTFGLRYGKRQAGTL------------KAQSNIW---------KI
GCC34347_1 Cpun 64 LVKREKDSASYNWNSFGLRYGKSLVDSE------------K----------------K
SOR24334_1 Aang 82 AAKKRENFSSYNWNSFGLRYGKR-----------------------------------
XP_015197899_1 Locu 111 AAKREKNLSAYNWNSFGLRYGKRRSNTP------------PPQL-------------G
XR_004403334_1 Pmar 98 -DADEKKGSTYNWNSFGLRFGKRELNFM-NISKILIIFTKR----------------Q
XP_015195061_1 Locu 59 GDSRRQAGEPYNVNSFGLRFGKKRDTQISWISSGLRSGGKRGRPERG---------EA
NP_001163986_1 Xtro 92 GRQKRQAQVGYNVNSFGLRFGKRATKLK-NRNSLKPQFGSKNQFSIPLNALRHAFCVE
consensus 211 ... . **.*.****.**. .

**Hsap**  REKDLPNYNWNSFGLRFGKR

**Oana**  QEKDLSAYNWNSFGLRYGKR

**Cmyd**  QEKDLSAYNWNSFGLRYGKR

**Xtro**  REKDLSTYNWNSFGLRYGKR

**Lcha**  RENDLSSYNWNTFGLRYGKR

**Locu**  REKNLSAYNWNSFGLRYGKR

**Aang**  KRENFSSYNWNSFGLRYGKR

**Cpun**  REKDSASYNWNSFGLRYGKS

**Xtro1b** KRQAQVGYNVNSFGLRFGKR

**Locu1b** RRQAGEPYNVNSFGLRFGKK

**Pmar**  DEKKGSTYNWNSFGLRFGKR

... . **.*.****.**.

>Kiss1Clust_NP_002247.3 metastasis-suppressor KiSS-1 preproprotein Homo sapiens

MNSLVSWQLLLFLCATHFGEPLEKVASVGNSRPTGQQLESLGLLAPGEQSLPCTERKPAATARLSRRGTSLSPPPESSGSPQQPGLSAPHSRQIPAPQGAVLVQREKDLPNYNWNSFGLRFGKREAAPGNHGRSAGRG

>Kiss1Clust_XP_028924153.1 metastasis-suppressor KiSS-1 Ornithorhynchus anatinus

MSSLTSLLLLLFLCAFPFGETVEKFTPIQNPRFSGRRSRQLASLLEQTPGLWEPVRERTRGQWERTPGQWERTPPCLDKKPGPEAAEQTARLALLCPPEESPGQVWRGLCPTQSQLVTGPQGGMLVEQEKDLSAYNWNSFGLRYGKRHAGVLKARLKIW

>Kiss1Clust_XP_027683240.2 metastasis-suppressor KiSS-1 Chelonia mydas

MASSGHVAGDLWLHHPLQEFFPSHLWISGSFGKYSTAEQGLGQPGMNPLSSMLLLLFFSNAPFGEPTERYLPLWSPRHTGDHFKHLASPAHWDQAIPCSESKPSPGKAEPKPVPPLLCKPQEESQAQLGQGIHPARSRAVSVPQGSPPMEQEKDLSAYNWNSFGLRYGKRQAAIGEAKSSHRA

>Kiss1Clust_NP_001156331.1 metastasis-suppressor KiSS-1 precursor Xenopus tropicalis

MSSLCLFLFLLGIHLGRSDHTAKNTDELYSQVPGKSQWLGSLLCPEKVPTTRRAEQMPVLSLLCRRKKSLSTGHPWSTDSLLPSRSISAPEGEFLVQREKDLSTYNWNSFGLRYGKRGSGSENSKTKVW

>Kiss1Clust_XP_005991747.1 PREDICTED: metastasis-suppressor KiSS-1 Latimeria chalumnae

MSPYIFLFLMLFLKGHLGEPLHTIVPIQTPVLTGEVLKAIASGLLQKEESAPCLVQTPQSRTQHPKMLRPLVKLFDLKHGSRPRISRKIGLSLCKFNSSSLGVQTVKRENDLSSYNWNTFGLRYGKRQAGTLKAQSNIWKI

>Kiss1Clust_SOR24334.1 Kisspeptin 1, partial Anguilla anguilla

SMVTIRFGETRSLRYTPYTADEDPEIAAMRVLRQMSEELSTEAPPYPHRPAHWPTALANPLPGGAGLPRRSWWWYPEVPPQAAKKRENFSSYNWNSFGLRYGKR

>Kiss1Clust_XP_015195061.1 PREDICTED_Lepisosteus oculatum

MRLPAISLLVAAVCLGVQSADPSQFLPLPLRTLRYRNVDSAPPPLPVDAPGLLASRLEGDSRRQAGEPYNVNSFGLRFGKKRDTQISWISSGLRSGGKRGRPERGEA

>Kiss1Clust_GCC34347.1 hypothetical protein Chiloscyllium punctatum

MNSEELHQAVGFPCSSHLRDPSVITNSKDPLYNLFRRQQALSWELCIIKLILNKHNSRGAELDLVKREKDSASYNWNSFGLRYGKSLVDSEKK

>Kiss1Clust_NP_001163986.1 kisspeptin 1b or 3 Xenopus tropicalis

MNLGGLSCFVICRLATDLQDFLTLFFIFFCNPEPTGGDGNLALTLDICDLIEVLYSSSTPQNFPTMPQVTTNTSQILKNYKEDLLASFFFQGRQKRQAQVGYNVNSFGLRFGKRATKLKNRNSLKPQFGSKNQFSIPLNALRHAFCVE

>Kiss1Clust_XP_015197899.1_PREDICTED_Lepisosteus oculatus

MGHTGKEMPCSPLRTESTMLLLTMMLMMSVQLVEPWAGHPQISSASPTVSGKKPEPGVQDILRRMSTTPPPGARLILPAAGKIPPALASLLFGSRFPRRGGWAQARPQPPAAKREKNLSAYNWNSFGLRYGKRRSNTPPPQLG

>Kiss1Clust_XR_004403334.1 PREDICTED: Petromyzon marinus uncharacterized LOC116945490

MRGLTVVTFLFLVLCCDSFGKVVSFYGFKESTKSGGGQLPGDVTDILREITSLLEGTDGIVAFYDFPGSGGSVDRAFMSPLHFYPMLRARMRSLPASDADEKKGSTYNWNSFGLRFGKRELNFMNISKILIIFTKRQ
