# Supplementary material for: Expression of Kisspeptin 1 in the Brain of the Adult Sea Lamprey Petromyzon marinus
Source: Life (Basel). 2021 Nov 3;11(11):1174. doi: 10.3390/life11111174 (PMC8624340; doi:10.3390/life11111174)
Supplement: Supplementary file 1 [file life-11-01174-s001.zip › life-1430285-supplementary for conversion/SupplementaryFile2.docx]

XP_032804800_1 Pmar MTPACSL-AALLAVCVFGGGAVAARTDRYGA-----------SPDSNHARRARSSEEIVTGDLRASPLRL
ACJ50540_1 Xtro MLLLLLL---TLVISQHAVGGTMFRGDEEGLELEEIGGPETSYPEGDPREKSESYELIPSADTLSWPGR-
XP_037771537_1 Cmid MTRLLLFSFIMVIIYQNGTFGKPVYGDLASTQLVDFSDAE--YPVSDTQAKRNSYQMSQDANSPNSAEQ-
ALD51308_1 Lcha MTRFLLF-FVMVIC-QNGAFGKHVYGNLPSNNQLEFSGRETLYPLSEAEAERQSHHMTRDIDAQNSAEH-
XP_015207582_1 Locu MNRFLLL-LAAVVVCQQRTMGKPLSGSSPAE---QFQNSAS----TPAGLGSLPHLAVREVEGPNSADQ-
SMN23033_1 Aang MSRFALL-LVFAVVCFCGAMGKSQNAFLSAE---RTDDSGSLYPERASAGVWDRSKVLREVTGPNYSDE-
GCC33732_1_Ki Cpun MTRLLLF-VVVIVNCHNAHLGKPISSE-------KFGSVDSMFPARQPQFSRNSPALARNLNSQNSPEQ-
XP_038666995_1 Scan MTKLLLF-LAVLVNFQNVYLGKPLTSE-------KFVSVDSMFPAREAPFPRNSHTLARSVDVQNSPQQ-
consensus *..... .......... ..... .. . . ..... .. ... ...... .....

XP_032804800_1 59 FGAVCRHAAETPRLLRLRALRGGHDLDAGLTDGEALPRSAEQDVTEFNYNPFGLRFGRRSGAQ-SSTAAT
ACJ50540_1 67 -SNICYFIREG-------------RLESQLSCHLRFTRS------KFNFNPFGLRFGKRARGD-ANGEGL
XP_037771537_1 68 -SSLCYFIQES-------------EIESQISCRLRFTRS------KFNFNPFGLRFGKRQQGSLASKRDP
ALD51308_1 68 -ASLCYFVQES-------------EIESQISCRLRFTRS------KFNFNPFGLRFGKRGQSS-SSNRNP
XP_015207582_1 62 -ASLCYFVQES-------------EVESQISCKLRFTRS------KFNFNPFGLRFGKRNRNI-VANDRS
SMN23033_1 66 -ADLCFFLKDS-------------EIEGHISCRLRYSRS------KFNRNPFGLRFGKREWSY-LPKSKT
GCC33732_1_Kiss 62 -SSLCYFLQES-------------DLESQISCKLRFTRS------KFNFNPFGLRFGKRGEAG-SVNWKA
XP_038666995_1 62 -PSMCYFLQES-------------DLESQISCKLRFTRS------KFNFNPFGLRFGKRDDAG-SVNWKT
consensus 71 ...*...... .............** .**.********.* . . . ..

XP_032804800_1 Pmar 128 RSRAEAACAPGKRGCRLVISKFKLRF-----------------
ACJ50540_1 Xtro 116 APLVPRRLLP-----------FLLKLKDKRCSE----SVGESC
XP_037771537_1 Cmid 118 ITLSSIKKLP-----------SLLKFKLNQMVPRCGDFGEQDC
ALD51308_1 Lcha 117 VLVSRKLIPQ-----------YLLKLKESRMLEF---YVSDGC
XP_015207582_1 Locu 111 AIPSEL--LL-----------YLLYLKETGLAP----------
SMN23033_1 Aang 115 AKPGTSKLLP-----------YLLYIQERKA------------
GCC33732_1_Kis Cpun 111 LTVNGNK-LP-----------YLWRH-----------------
XP_038666995_1 Scan 111 LVVNGNK-LP-----------YLWKHKVPT-------------
consensus 141 . . . .. .......

**Cmyd**  KFNFNPFGLRFGKRQQGS

**Xtro**  KFNFNPFGLRFGKRARGD

**Lcha**  KFNFNPFGLRFGKRGQSS

**Locu**  KFNFNPFGLRFGKRNRNI

**Aang**  KFNRNPFGLRFGKREWSY

**Cpun**  KFNFNPFGLRFGKRGEAG

**Scan**  KFNFNPFGLRFGKRDDAG

**Pmar**  EFNYNPFGLRFGRRSGAQ

.**.********.* .

>Kiss2Cluster_GCC33732.1_Kiss2_Chiloscyllium punctatum

MTRLLLFVVVIVNCHNAHLGKPISSEKFGSVDSMFPARQPQFSRNSPALARNLNSQNSPEQSSLCYFLQESDLESQISCKLRFTRSKFNFNPFGLRFGKRGEAGSVNWKALTVNGNKLPYLWRH

>Kiss2Cluster_ACJ50540.1 kisspeptin2_Xenopus tropicalis

MLLLLLLTLVISQHAVGGTMFRGDEEGLELEEIGGPETSYPEGDPREKSESYELIPSADTLSWPGRSNICYFIREGRLESQLSCHLRFTRSKFNFNPFGLRFGKRARGDANGEGLAPLVPRRLLPFLLKLKDKRCSESVGESC

>Kiss2Cluster_ALD51308.1 kisspeptin_Latimeria chalumnae

MTRFLLFFVMVICQNGAFGKHVYGNLPSNNQLEFSGRETLYPLSEAEAERQSHHMTRDIDAQNSAEHASLCYFVQESEIESQISCRLRFTRSKFNFNPFGLRFGKRGQSSSSNRNPVLVSRKLIPQYLLKLKESRMLEFYVSDGC

>Kiss2Cluster_XP_015207582.1 PREDICTED: uncharacterized protein LOC107077970_Lepisosteus oculatus

MNRFLLLLAAVVVCQQRTMGKPLSGSSPAEQFQNSASTPAGLGSLPHLAVREVEGPNSADQASLCYFVQESEVESQISCKLRFTRSKFNFNPFGLRFGKRNRNIVANDRSAIPSELLLYLLYLKETGLAP

>Kiss2Cluster_SMN23033.1 Kisspeptin 2 precursor Anguilla anguilla

MSRFALLLVFAVVCFCGAMGKSQNAFLSAERTDDSGSLYPERASAGVWDRSKVLREVTGPNYSDEADLCFFLKDSEIEGHISCRLRYSRSKFNRNPFGLRFGKREWSYLPKSKTAKPGTSKLLPYLLYIQERKA

>Kiss2Cluster_XP_037771537.1 uncharacterized protein LOC102940483 KISS2_Chelonia mydas

MTRLLLFSFIMVIIYQNGTFGKPVYGDLASTQLVDFSDAEYPVSDTQAKRNSYQMSQDANSPNSAEQSSLCYFIQESEIESQISCRLRFTRSKFNFNPFGLRFGKRQQGSLASKRDPITLSSIKKLPSLLKFKLNQMVPRCGDFGEQDC

>Kiss2Cluster_XP_038666995.1 kisspeptin 2_Scyliorhinus canicula

MTKLLLFLAVLVNFQNVYLGKPLTSEKFVSVDSMFPAREAPFPRNSHTLARSVDVQNSPQQPSMCYFLQESDLESQISCKLRFTRSKFNFNPFGLRFGKRDDAGSVNWKTLVVNGNKLPYLWKHKVPT

>XP_032804800.1 uncharacterized protein LOC116939896 [Petromyzon marinus] LAMPREY_Kiss2pot

MTPACSLAALLAVCVFGGGAVAARTDRYGASPDSNHARRARSSEEIVTGDLRASPLRLFGAVCRHAAETPRLLRLRALRGGHDLDAGLTDGEALPRSAEQDVTEFNYNPFGLRFGRRSGAQSSTAATRSRAEAACAPGKRGCRLVISKFKLRF
